# Effects of Simulated Short‐Haul Flights on Sport‐Specific Performance, Sleep, and Psychophysiological Responses in Football Players

**DOI:** 10.1111/sms.70169

**Published:** 2025-11-18

**Authors:** Dimitris C. Stergiopoulos, Petros G. Botonis, Panagiotis G. Miliotis, Athanasios X. Zavvos, Spiridoula D. Ntalapera, Georgios L. Papaleontiou, Giannis A. Pilatis, Evangelia K. Soukara, Nickos D. Geladas

**Affiliations:** ^1^ School of Physical Education and Sports Science National and Kapodistrian University of Athens Athens Greece; ^2^ Institute for Sports Performance, Biorhythms, and Environment National and Kapodistrian University of Athens Athens Greece; ^3^ Center for Aviation Medicine Athens Greece

**Keywords:** air travel, fatigue, football, sleep, sport‐specific performance, team sports

## Abstract

The effects of two short‐haul air travel (SHAT) of different durations on sport‐specific performance, sleep, and psychophysiological indices in highly trained football players were examined. Nineteen participants completed, in a randomized order, two simulated SHAT, 1 week apart, each lasting 4 h and 2 h, followed by a control condition (CON). Generic (10‐m sprint, repeated sprint ability; RSA, countermovement jump; CMJ), sport‐specific performance (Hoff Test, Loughborough Soccer Passing Test; LSPT), and psychophysiological responses (sleep, heart rate variability [HRV], salivary cortisol and immunoglobulin‐A [sIgA], and perceptual recovery) were evaluated before (Baseline) and after each condition. Compared to Baseline, RSA and 10‐m sprint declined by 3.5% and 6%, respectively, on the day after travel (D + 1) (*p* < 0.001 and *p* < 0.01). Likewise, CMJ and total distance covered during the Hoff test decreased by 5.5% at D + 1 (*p* < 0.001). LSPT performance decreased only after the 4 h flight (*p* < 0.05). Total sleep time was reduced the night before SHAT (*p* < 0.001) compared to Baseline. sIgA was reduced on the day of the travel (DT) and D + 1 after both SHAT, while HRV was decreased at DT only after the 4 h SHAT (*p* < 0.05). Mood states remained unaffected by travel, regardless of duration. We currently suggest that short‐haul air travel, whether 4 or 2 h, induces psychophysiological perturbations and performance deterioration, likely due to the travel restrictive conditions and the inadequate sleep obtained the night before the flight. Since both flights were conducted early in the morning, more research is warranted to investigate the role of departure time on football performance.

## 1 Introduction

Elite football players frequently undertake short‐haul air travel (< 5 h), throughout the season. For instance, Australian football teams have been reported to travel 26 times covering up to 3500 km in each flight [1]. Elite European football clubs, participating in important international competitions (UEFA Champions League, UEFA Europa League, and UEFA Conference League) are expected to engage in a comparable number of short‐haul air flights.

Travel‐related stressors such as disruptions to individual routines and restricted activity, along with exposure to conditions equivalent to a FiO_2_ of 0.16–0.18 at sea level, due to the reduced cabin pressure during flight, may disturb travelers' well‐being and increase perceived fatigue [2, 3, 4]. So far, research studies investigating the influence of short‐haul traveling (2–5 h) on sleep and perceived fatigue/recovery status in athletes or physically active individuals have shown inconsistent results [5, 6, 7]. McGuckin et al. found reduced alertness and increased stress following a 3‐h flight [5]. In contrast, Fowler et al. reported that a 5‐h flight had no effect on sleep and perceived recovery in football players [6]. A more recent study also showed that a simulated 5‐h travel increased muscle soreness, but had no effect on sleep‐related measures of physically active individuals [7]. Notwithstanding the potential well‐being deterioration, a common finding across these studies is that physical tests requiring intermittent endurance, strength, and jumping ability [5, 7] remain unaltered post‐flight, suggesting that athletic or competition performance is not compromised post‐travel.

Despite the consensus that short‐haul air travel has a negligible effect on exercise performance, we cannot ignore important factors that may have affected the existing findings. These include: (i) fitness background of participants, (ii) the type and sensitivity of testing, and (iii) experimental design. Besides, many of the studies conducted so far have overlooked a crucial aspect of the actual day‐to‐day schedules of football players. Understanding the real‐life contributors to air travel fatigue in football‐specific settings (departure time and air travel duration, busy calendar, individual routines, etc.) is important. Additionally, some of the tests previously applied were not football‐specific. While the application of generic tests (e.g., handgrip strength and jump) may provide significant information and assist with testing logistics, their ecological validity in football may be limited.

Moreover, no study has yet examined the influence of different flight durations on football‐specific performance and football players' perceived fatigue/recovery status. This is particularly relevant in real‐life scenarios, where elite football players frequently travel to different European destinations with short (e.g., 2‐h) or long (e.g., 4‐h) flights. It is expected that longer flights (e.g., 4 h) would impose greater psychophysiological stress on football players compared to shorter flights. To this end, only one study [7] has considered important physiological (i.e., sleep, hormonal responses, oxygen saturation, heart rate) responses that may influence performance and perception following travel. Given these literature gaps, the impact of short‐haul air travel of different flying durations on sport‐specific performance and psychophysiological responses of football players warrants further investigation.

Furthermore, our understanding of the impact of short‐haul flights on the cardiovascular system and immune function is limited. Considering the recovery process from a demanding match after short‐haul travel, the role of vagal tone and the risk of upper respiratory tract infection may be crucial for many athletes who routinely travel for athletic competitions. Thus far, no studies have examined the effects of short‐haul air travel on heart rate variability and immune markers in competitive real‐life football settings.

Namely, nowadays there is extensive short‐haul traveling in elite soccer players who are also obliged to participate in regular high‐intensity training and quite often in two official games per week. On the contrary, only one piece of research regarding short‐haul flights and performance exists, neglecting the possible deleterious effects of mild hypoxia, somatic inertia during travel, and disruption of sleep patterns in an additive or interactive manner. Potentially, this lack of information has paramount importance for travel logistics in elite athletes.

This study aimed to examine the effects of two simulated short‐haul air travels of different durations on sport‐specific performance, sleep, and recovery indices in football players within a competitive schedule. It was anticipated that compared to a control condition (no travel), short‐haul air travel, irrespective of duration, would have a negative influence on the psychophysiological responses of footballers thus, leading to sport‐specific performance deterioration. It was further expected that compared to a shorter flying duration (i.e., 2 h), longer flights (i.e., 4 h) would exacerbate travel fatigue, resulting in greater post‐travel performance deterioration.

## 2 Methods

### 2.1 Participants

Twenty‐two high‐level male football players were screened, nineteen of whom (age: 21.3 ± 4.2 years, height: 179.6 ± 6.6 cm, body mass: 73.2 ± 9.1 kg, fat mass: 11.9% ± 3.1%, body mass index: 22.7 ± 2.1, Yo‐Yo Intermittent Recovery Test level 1‐YYIR1: 1823.2 ± 324.8 m) volunteered to participate in the present study. Three participants were excluded based on the following criteria: illness, injury, and being unable to complete the entire simulation flights. All participants were non‐smokers and were free of any cardiac, respiratory, or musculoskeletal disorders. They were informed in detail about the purpose of the study, the experimental procedures, and the potential risks involved, before giving their informed consent for participation. The participants were instructed to avoid consuming alcohol 3 days before testing and remain fasted for 3 h before the exercise protocol. Nutritional intake and hydration status were documented 24 h before the first trial and were replicated for all subsequent trials. The experimental procedure is being performed by the Declaration of Helsinki and was approved by the Ethics Committee of the Kapodistrian University of Athens, School of Physical Education and Sports Science (1274/17‐03‐2021).

### 2.2 Experimental Design

**FIGURE 1:**
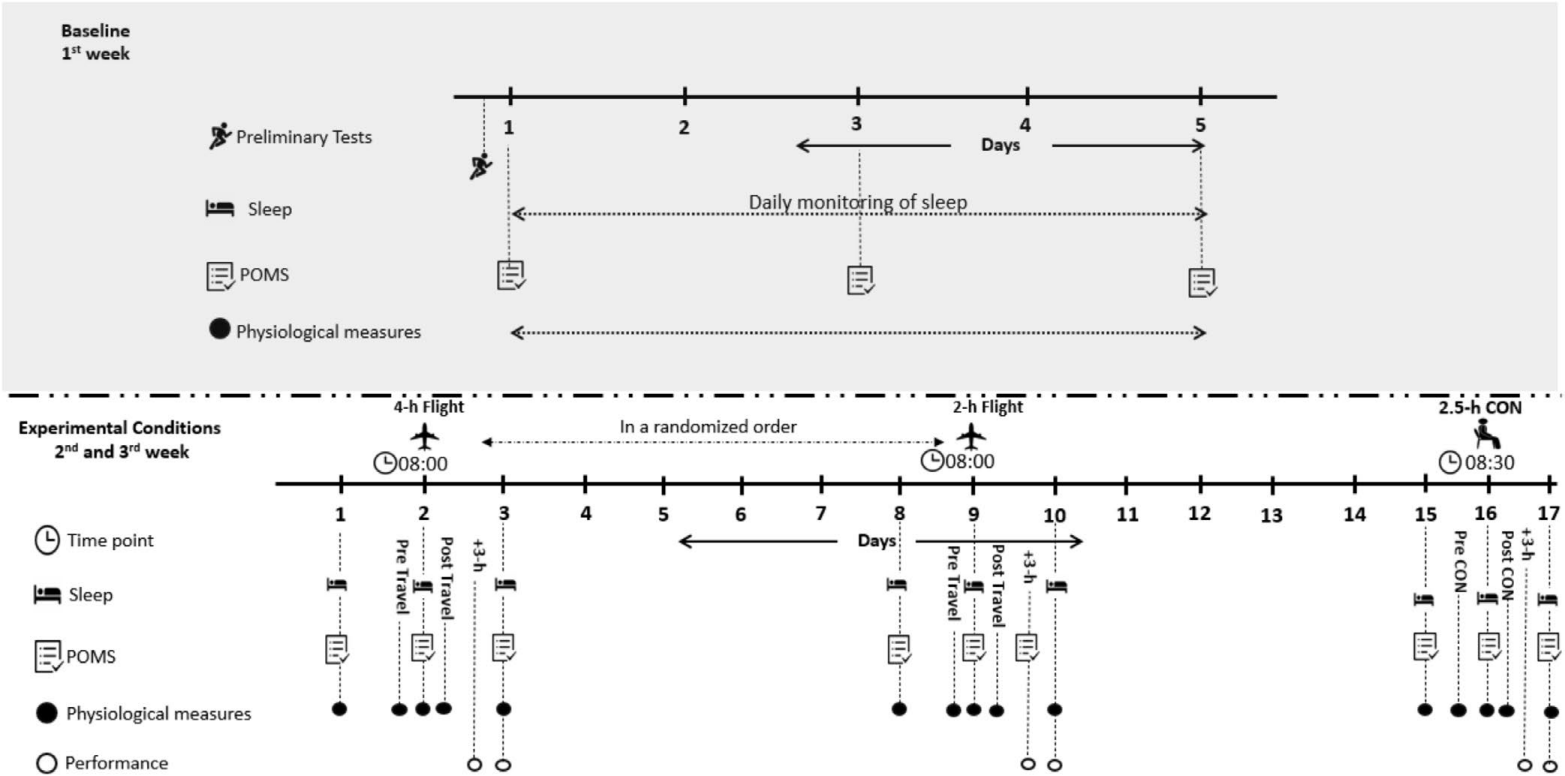
Schematic outline of the study design illustrating performance, physiological, perceptual, and training data collection and time points; Preliminary tests and Baseline measurements (BAS), two short‐haul air travels (4‐h, 2‐h long), non‐simulated air travel (CON), +3‐h: 3 h following the trial, and Profile of Mood States (POMS).

Following familiarization with all measures and procedures, three experimental conditions were conducted. Initial trials involved completing two simulated short‐haul air travels (SHAT), 1 week apart, each lasting 4 and 2 h, in a randomized order, followed by a control condition. The simulated flights represented typical international air travel of elite European football clubs. A control trial (CON), was completed requiring the players to stay still in the laboratory at sea level. Physiological, performance, and perceptual data were collected a week before (baseline) and at standardized time points throughout the experimental conditions. The study design is illustrated in Figure 1.

### 2.3 Simulated Travel

The participants completed two simulated air travels (4‐h and 2‐h long) a week apart. Altitude simulation was accomplished using a hypobaric chamber (Environmental Tectonics Corporation, 1978) at the Center for Aviation Medicine in the 251 Hellenic Air Force General Hospital. The barometric pressure in the hypobaric chamber was adjusted at a rate of 8.000 ft simulating 2.438 m of geographical altitude. This altitude corresponds to an internal barometric pressure of ~564.5 mmHg which implies a partial pressure of Oxygen equal to 119 mmHg, equivalent to 16% inspired Oxygen at sea level. Seating adjustments were made to replicate typical conditions experienced during a commercial flight. Activity and lighting were regulated to mimic the behavior of passengers during the actual airline travel. Participants were served meals and beverages according to the schedule of a typical commercial flight. Specifically, during the simulated SHAT or Control trials, players' food intake included a cereal bar, a small sandwich with chicken and vegetables, and 250 mL of orange juice.

### 2.4 Experimental Procedures

#### 2.4.1 Preliminary Tests

Before evaluating physical performance, a 10‐min warm‐up was administered consisting of 5 min jogging at 8.0 km.h^−1^ and 5 min stretching. To assess the power of lower extremities and sprint performance, participants completed a countermovement jump (CMJ) and a 10 m sprint. In another visit, we used both the YYIR1 [8] and the Hoff test [9] to evaluate football‐specific aerobic capacity. All testing was done at an outdoor artificial football pitch in temperate conditions (Temperature: 25°C ± 3°C and Humidity: 8% ± 6%).

#### 2.4.2 Physical Performance and Football‐Specific Tests

Participants were tested at standardized time points. CMJ (Optogait, Microgate, Bolzano, Italy) was evaluated at three different times: baseline, 3 h after the flight (+3‐h), and 1 day after (D + 1) the conditions SHAT or CON. Sprint performance at 10 m, repeated sprint ability (RAST) (Microgate, Bolzano, Italy), and the Hoff test were assessed at baseline and D + 1 after each trial. The Hoff test has been suggested as a valid alternative to field tests for assessing specific endurance in football [9]. During the circuit test, players need to dribble the ball and cover as much distance as possible within a 10‐min period. Blood lactate levels were measured before and 3 min after the Hoff test (Lactate Scout Senslab, Germany). Previous studies have found a strong link between aerobic capacity and performance in the Hoff test [10]. The Loughborough Soccer Passing Test (LSPT) was carried out at baseline, 3 h after, and on the day after each trial. The test assesses specific football skills such as passing ability, dribbling, ball control, and decision‐making [11].

### 2.5 Physiological Measurements

#### 2.5.1 Sleep

Sleep was monitored using commercially available accelerometers (GT9X, ActiGraph) and analyzed using the manufacturer's software (version 6.13.3, ActiGraph). The participants put on the accelerometers before their nighttime sleep on their nondominant wrist and removed them upon awakening. The participants were asked to complete a diary before going to bed and upon awakening each morning in which they noted time in bed and wake‐up time. The self‐reported data were entered into ActiLife software. The Cole‐Kripke automatic sleep/wake algorithm was used to score sleep wherein sleep is classified based upon activity levels and a sleep/wake calculation. This algorithm has been previously validated to classify sleep in adults [12]. The activity counts were collected in 1‐min epochs, and the sampling frequency was set at 30 Hz. The following dependent variables were derived from sleep diary and activity monitor data: (1) total sleep time (in hours: minutes; the amount of time spent in bed asleep), (2) sleep onset latency (min), (3) sleep efficiency (%, sleep duration expressed as a percentage of time in bed), (4) number of awakenings (*n*), and (5) duration of awakenings (min). The accelerometers presently used for sleep assessment have been validated against polysomnography and have shown high sensitivity and accuracy (intraclass correlation coefficient: 0.83–0.96). Sleep patterns were assessed throughout the experimental sessions at the same time points: 1 day before (D−1), the day of the simulated flight (DF) or CON (DC), and the following day (D + 1) [13].

#### 2.5.2 Cardiovascular, Saliva, and Hydration Measures

The assessment of heart rate variability (HRV) was conducted in a quiet, calm setting at baseline (5 days), D−1, DF or DC, and D + 1 at the same times in the afternoon (15:00 EET) before the training session to minimize any variations in response due to circadian rhythm [14]. The R‐R intervals were continuously recorded in the sitting position for 5 min using a portable heart rate monitor, Polar V800 (Polar Electro Inc., Bethpage, NY, USA). Afterward, the heart rate data were transferred to a computer using Bluetooth technology for further processing and analysis. HRV data processing was performed using the Kubios HRV analysis software (MATLAB, version 3.3.1, Kuopio, Finland). The signal quality was visually inspected, and if there were 10% ectopic beats, the data were discarded to prevent contamination. HRV was analyzed using linear statistical measures (time domain) and specifically the logarithm of the square root of successive mean squared differences of R‐R (LnRMSSD) in milliseconds, representing parasympathetic modulation.

Salivary cortisol and immunoglobulin (sIgA) measures were obtained from all players via passive drool in the morning (between 07:00 and 08:00 EET) at the same time point (D−1, DF or DC, and D + 1) for each trial. The saliva collection protocol required participants not to have eaten food, drank water, and refrained from brushing their teeth. Players placed the oral fluid collector (OFC) swab (Soma Bioscience) on their tongue and closed their mouth. They did not suck or move the swab around their mouth to ensure the test was consistent and reduced variability [15]. The indicator on the stem turned blue when the sample was complete (swab collected 0.5‐mL oral fluid). The swab was then placed in the OFC buffer bottle of assays (sodium phosphate, salts, detergents, and preservatives). Thereafter, the samples were gently mixed in the OFC buffer bottle for 2 min. Two drops of the sample were added to the sample window of the lateral flow device and left for 15 min (“incubation” phase). The strip was placed in the lateral flow device real‐time reader with results ready within approximately 20 s. It has been demonstrated that the lateral flow device has high reliability (ICC: 0.868) and validity against the enzyme‐linked immunosorbent assay method (*p* = 0.881) [16].

Hemoglobin oxygen saturation (SpO_2_%) and heart rate were recorded with the participants seated using a pulse oximeter (Nonin 9560, Bluetooth Pulse Oximeter, Nonin, North Plymouth, Minnesota, USA) for 5 min immediately prior to, and every 30 min throughout the experimental conditions. To assess the hydration status, body weight (Seca 762, Germany), and urine specific gravity (USG) were collected immediately before and after each trial (Siemens Multistix 10 SG Urine Reagent Test Strips).

### 2.6 Perceptual Measures

The Profile of Mood States‐Short form [17] was filled at baseline, D−1, DF or DC, and D + 1 at the same times in the afternoon (07:00 PM Eastern European Time, EET). Daytime sleepiness was quantified using the Stanford Sleepiness Scale [18].

### 2.7 Statistical Analysis

All results are expressed as mean ± standard deviation. Statistical power was calculated using G*power 3.1.9.4. The assumption of normality was verified using the Shapiro–Wilk test. Linear mixed models were performed for each dependent variable (performance metrics, sleep, HRV, sIgA, and mood states) using “conditions” (baseline, simulated flights [4 and 2 h] as fixed effect, and the control trial) and “subjects” as a random effect. Bonferroni adjustment was used to identify differences between pairwise comparisons. As a measure of effect size, Cohen's *d* was calculated, and values of 0.20, 0.50, and above 0.80 were considered as small, medium, and large, respectively [19]. The level of significance was set at *p* < 0.05. Analyses were performed using IBM SPSS 20.0.0 (Statistical Package for Social Sciences, SPSS Inc., Chicago, IL, USA).

## 3 Results

### 3.1 Football‐Specific Tests, Technical Skills, and Physical Performance

The total distance covered in the Hoff test was different among conditions (main effect: *p* < 0.001) (Table 1). Notably, compared with baseline and regardless of air travel duration, the total distance covered in the Hoff test was shorter both following 4‐h (−5%) and 2‐h (−6%) of air travel (*p* = 0.02; *d* = −1.12, and *p* < 0.01; *d* = −0.97, respectively), whilst no differences were shown between baseline and control trial (*p* > 0.05).

**TABLE 1:** Performance in the Hoff test during baseline, and following experimental trials. Values are presented as Mean ± SD.

*N* = 19	Baseline	4‐h flight	2‐h flight	Control	*p* value (Main Effect)
Hoff_Distamce covered_ (m)	1782.5 ± 134.8	1686.8 ± 150.9^b^	1669.8 ± 178.5^b^	1773.9 ± 144.9	*p* < 0.001
BLa (mmol.l^−1^)	8.4 ± 2.3	6.8 ± 2.6^a^	6.2 ± 2.1^c^	7.1 ± 2.6	*p* < 0.001
HR_avg_ (beats.min^**−1**^)	172 ± 11	172 ± 11	169 ± 12	166 ± 11	*p* < 0.001
HR_peak_ (beats.min^**−1**^)	187 ± 9	185 ± 9	183 ± 11^a^	185 ± 10	*p* < 0.001

*Note:* Main effect refers to the conditions (Baseline, 4‐h flight, 2‐h flight, Control).

Abbreviations: BLa, blood lactate concentration; Hoff_Distance covered_, distance covered during Hoff test; HR_avg_, average heart rate; HR_Max_, maximum heart rate.

^a^ Significantly different from Baseline (*p* = 0.01).

^b^ Significantly different from Baseline and Control trial (*p* < 0.01).

^c^ Significantly different from Baseline (*p* < 0.001).

As regards the exercise intensity during the Hoff test, significant differences were found in peak and average heart rate (HR_peak_. HR_avg_) among conditions (main effect: *p* < 0.01). HR_peak_ was decreased only after 2‐h air travel (182.5 ± 6.8 beats.min^**−1**^) compared with baseline (186.9 ± 8.5 beats.min^−1^, *p* = 0.01; *d* = −0.31). Additionally, the post hoc comparison revealed similar HR_avg_ among conditions (*p* > 0.05). Blood lactate measured 3 min after the effort was also significantly different among conditions (main effect: *p* < 0.001). Compared with baseline (8.4 ± 2.3 mmol.l^−1^), blood lactate was decreased following short‐haul air travels (4‐h: 6.8 ± 2.6 mmol.l^−1^, *p* = 0.01, *d* = −0.74; 2‐h: 6.2 ± 2.1 mmol.l^−1^, *p* < 0.001, *d* = −1.52).

The analysis of variance revealed significant differences among conditions (main effect: *p* < 0.001) in 10‐m sprint performance. Specifically, regardless of air travel duration and when compared with baseline measurements, 10‐m sprint performance decreased by 6% (1.74 ± 0.09 s vs. 1.84 ± 0.12 s, *p* < 0.001). There were no significant differences between 4‐h and 2‐h simulated travels (1.84 ± 0.13 s vs. 1.84 ± 0.11 s, *p* > 0.05). Similarly, sprint performance showed no significant difference between baseline and control conditions (1.74 ± 0.09 s vs. 1.77 ± 0.09 s, *p* > 0.05).

RAST results are shown in Table 2. Significant differences were observed among experimental conditions (main effect: *p* < 0.001). Compared with baseline and control trials, mean, total, and best time in RAST were increased by ~3.5% following both simulated flights (*p* < 0.001). However, the percentage sprint decrement was similar among conditions (*p* = 0.67). CMJ deteriorated after simulated flights (*p* < 0.001), and no interaction was observed between condition × time (*p* = 0.84). Specifically, regardless of air travel duration, CMJ decreased by approximately 8.5% after 3‐h and by approximately 5.5% at D + 1, compared with baseline (*p* < 0.001) (Table 3).

**TABLE 2:** Running anaerobic sprint test (RAST, 4 × 35 m) values at baseline, and following experimental trials. Values are presented as Mean ± SD.

*N* = 19	Baseline	4‐h flight	2‐h flight	Control	*p* value main effect
Mean time (s)	5.21 ± 0.26	5.35 ± 0.31^d^	5.41 ± 0.30^e^	5.21 ± 0.23	*p* < 0.001
Total time (s)	31.24 ± 1.56	32.11 ± 1.87^d^	32.44 ± 1.79^e^	31.25 ± 1.37	*p* < 0.001
Best time (s)	4.83 ± 0.15	4.95 ± 0.24^a^	5.01 ± 0.28^b^, ^c^	4.85 ± 0.15	*p* < 0.001
Sprint decrement (%)	7.8 ± 3.3	8.2 ± 2.3	8.0 ± 2.1	7.4 ± 2.5	*p* = 0.671

*Note:* Main effect refers to the conditions (Baseline, 4‐h flight, 2‐h flight, Control).

^a^ Significantly different from Baseline (*p* < 0.05).

^b^ Significantly different from Baseline (*p* = 0.001).

^c^ Significantly different from Control (*p* < 0.01).

^d^ Significantly different from Βaseline and Control trials (*p* < 0.01).

^e^ Significantly different from Baseline and Control trials (*p* < 0.001).

**TABLE 3:** LSPT and CMJ values at baseline, +3‐h, and D + 1 following experimental trials. Values are presented as Mean ± SD.

*N* = 19	Baseline	4‐h flight		2‐h flight		Control		*p* value condition	*p* value time	Condition × Time
		+3‐h	D + 1	+3‐h	D + 1	+3‐h	D + 1			
LSPT_Time taken_ (s)	38.7 ± 2.9	39.7 ± 2.9	39.5 ± 2.5	40.2 ± 2.6^a^	38.6 ± 3.0	39.8 ± 3.4	38.8 ± 2.2	*p* = 0.72	*p* = 0.02	*p* = 0.34
LSPT_Penalty_ (s)	1.2 ± 5.4	4.5 ± 4.5^b^, ^d^	4.9 ± 7.5^b^, ^d^	3.9 ± 5.2	1.1 ± 4.1	0.6 ± 4.5	3.1 ± 5.2	*p* = 0.01	*p* > 0.05	*p* = 0.03
LSPT_Performance_ (s)	39.8 ± 5.4	44.2 ± 5.1^c^, ^d^	44.4 ± 6.8^c^, ^d^	44.1 ± 6.3	39.7 ± 5.0	40.3 ± 5.7	41.8 ± 5.5	*p* = 0.02	*p* = 0.32	*p* = 0.03
CMJ (cm)	38.8 ± 4.6	35.3 ± 5.0^e^	36.4 ± 4.9^e^	35.6 ± 5.5^e^	36.8 ± 5.6^e^	37.1 ± 5.2^c^	37.8 ± 4.8	*p* < 0.001	*p* < 0.01	*p* = 0.84

*Note:* Condition refers to Baseline, 4‐h flight, 2‐h flight, and Control. Time refers to +3‐h and D + 1.

Abbreviations: CMJ, countermovement jump; LSPT, Loughborough soccer passing test.

^a^ Significantly different from D + 1 (*p* < 0.05).

^b^ Significantly different from Baseline (*p* < 0.05).

^c^ Significantly different from Baseline (*p* = 0.01).

^d^ Significantly different from Control (*p* < 0.05).

^e^ Significantly different from Baseline (*p* < 0.001).

Performance scores for LSPT across conditions are presented in Table 3. Performance in LSPT was decreased after the 4‐h short‐haul flight by approximately 11% compared with the baseline and control trials (*p* < 0.05), with an interaction between condition × time (*p* = 0.03). Similarly, penalty time (LSPT_Penalty_) was impaired after the 4‐h air travel (*p* < 0.05), with an interaction between condition × time (*p* = 0.03). No significant differences were observed among conditions in the time required to complete the test (LSPT_Time taken_) (*p* > 0.05).

### 3.2 Sleep

**FIGURE 2:**
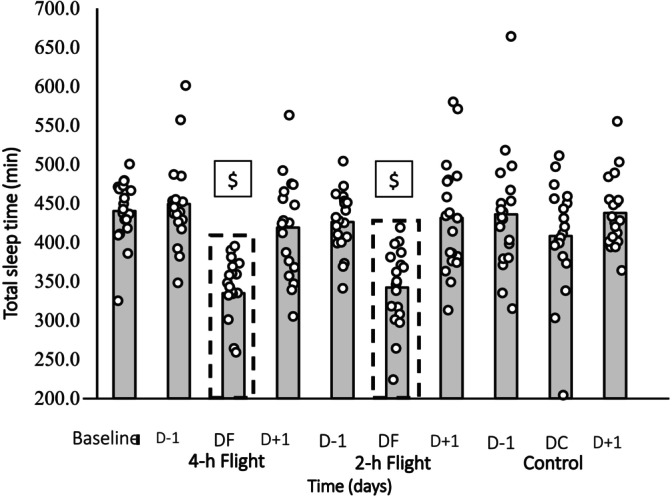
The figure shows the individual data of the participants' sleep duration at baseline, simulated air travel (4‐h, 2‐h), and control trial (*N* = 19). The thick dotted black boxes highlight the night before the flights. The gray bars show the mean value. D−1: 1 day prior to the flight; DF: Day of flight; D + 1: 1 day following the flight. ^$^Significant different from Baseline and Control trials (*p* < 0.01).

Total sleep time was reduced by 114 and 107 min the night before the 4‐h and 2‐h flights (*p* < 0.01, *d* = 1.22 and *d* = 1.46, respectively). A substantial decrease in total sleep time was observed the night before the simulated flights, regardless of flight duration, compared to baseline and control trials (*p* < 0.01), with a significant interaction between condition and time (*p* < 0.001). The total sleep time was similar before the 4‐h and 2‐h flights, with no significant differences in sleep efficiency, number of awakening events, or duration of awakenings between baseline and control (*p* > 0.05) (Table 4). In addition, Figure 2 illustrates all the individual cases for sleep duration.

**TABLE 4:** Objective sleep patterns at baseline and experimental conditions. Values are presented as Mean ± SD.

*N* = 19	Baseline	4‐h flight			2‐h flight			Control			*p* value condition main effect	*p* value time main effect	Condition × Time
		D−1	DF	D + 1	D−1	DF	D + 1	D−1	DC	D + 1			
Total sleep time (min)	440.3 ± 40.3	449.1 ± 56.7	334.9 ± 51.9^**$**^	418.9 ± 64.5	426.2 ± 39.5	342.1 ± 49.6^a^	431.5 ± 71.9	435.9 ± 76.4	408.2 ± 71.2	437.8 ± 45.8	*p* < 0.001	*p* < 0.001	*p* < 0.001
Sleep latency (min)	9.3 ± 5.3	7.7 ± 5.8	7.5 ± 5.0	5.5 ± 3.7	6.9 ± 4.9	6.4 ± 5.1	7.4 ± 5.1	7.5 ± 3.2	8.5 ± 4.7	5.8 ± 4.0	*p* = 0.89	*p* = 0.27	*p* = 0.39
Sleep efficiency (%)	87.4 ± 4.2	88.6 ± 4.3	88.0 ± 4.8	89.0 ± 5.2	88.3 ± 4.5	88.3 ± 4.4	87.9 ± 3.7	88.9 ± 4.4	85.7 ± 6.7	89.8 ± 2.5	*p* = 0.79	*p* = 0.09	*p* = 0.27
Awakenings (n)	17.6 ± 7.7	15.9 ± 8.2	13.5 ± 5.2	13.9 ± 4.7	16.6 ± 7.9	13.2 ± 6.4	14.6 ± 7.7	16.2 ± 7.8	15.5 ± 7.8	16.2 ± 6.7	*p* = 0.33	*p* = 0.14	*p* = 0.84
Duration of awakenings (min)	2.5 ± 0.5	2.4 ± 0.6	2.8 ± 1.6	2.8 ± 1.1	2.6 ± 0.7	2.2 ± 0.4	2.8 ± 1.1	2.8 ± 0.7	2.4 ± 0.9	2.5 ± 0.7	*p* = 0.38	*p* = 0.33	*p* = 0.12

*Note:* Main effect refers to the conditions (Baseline, 4‐h flight, 2‐h flight, Control). Condition refers to Baseline, 4‐h flight, 2‐h flight, and Control. Time refers to D−1, DF or DC, and D + 1.

Abbreviations: D + 1, 1 day following the flight; D−1, 1 day prior to the flight; DC, day of control; DF, day of flight.

^a^ Significant difference from Baseline and Control trials (*p* < 0.01).

### 3.3 Cardiovascular, Saliva, and Hydration Measures

**FIGURE 3:**
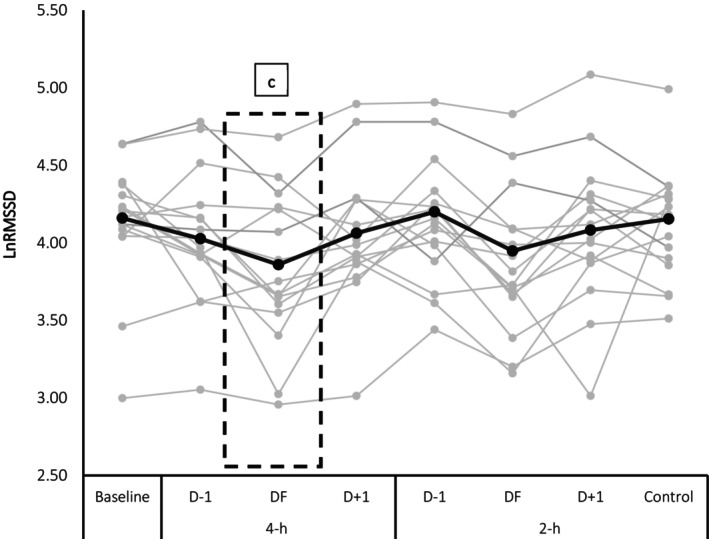
Mean LnRMSSD (thick black line and black circles) and individual responses (gray line and gray circles) (*N* = 19). LnRMSSD was measured during baseline, air travels (4‐h, 2‐h), and control trials. The thick dotted black box highlights the day with significant differences. D−1: 1 day prior to the flight; DF: Day of flight; D + 1: 1 day following the flight. ^c^Indicates significant difference from Baseline and D−1 (*p* < 0.05).

Regardless of air travel duration, SpO_2_% and heart rate were significantly different compared with the control trial (*p* < 0.01). The mean range of SpO_2_% and heart rate at sea level was 98% ± 0.8% and 64 ± 0.6 beats.min^−1^, respectively and during the flight corresponded to 93% ± 0.3% and 57 ± 1.4 beats.min^−1^, respectively. LnRMSSD values are shown in Figure 3. Compared to Baseline and D−1, LnRMSSD was decreased only on the day of 4‐h travel (DF) (*p* < 0.05; *d* = −0.64), and no interaction was observed between condition × time (*p* = 0.39).

**FIGURE 4:**
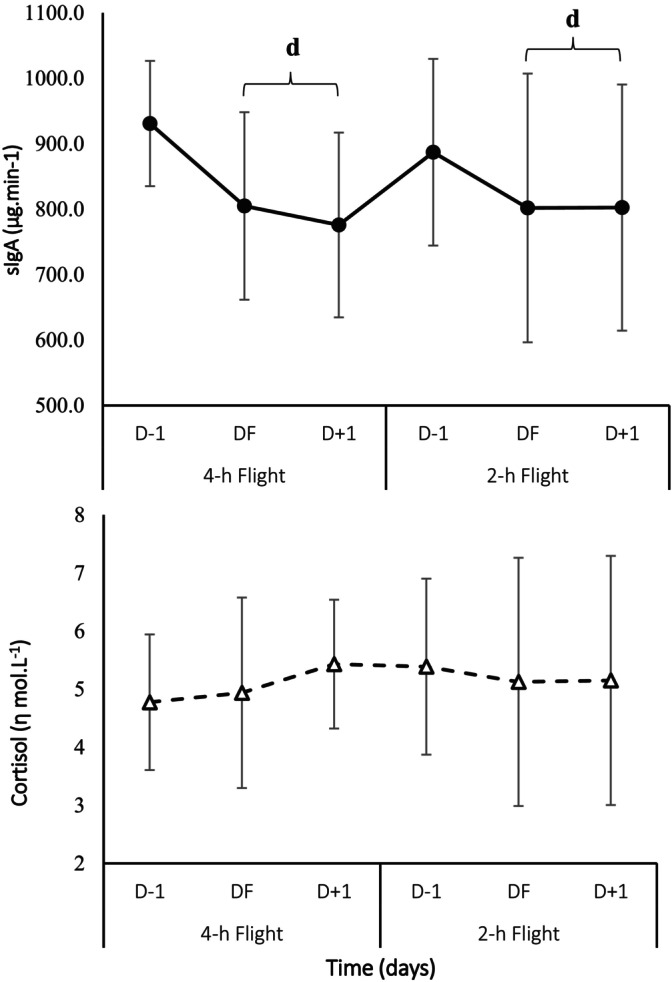
Secretory sIgA (a) and salivary cortisol (b) concentrations at D−1, DT, and D + 1 of simulated air travel (4‐h, 2‐h). Values are presented as Mean ± SD. (*N* = 16). D−1: 1 day prior to the flight; DF: Day of flight; D + 1: 1 day following the flight. ^d^Indicates significant difference from D−1 (*p* < 0.05). Condition × Time, *p* > 0.05.

Secretory immunoglobulin A (sIgA) was decreased similarly in both flights on the day of flight (DF) and the following day (D + 1) compared to the day prior (D−1) (*p* < 0.05; *d* = 0.95) (Figure 4). No significant differences were observed between simulated flights for salivary cortisol (*p* > 0.05). No significant differences were observed among experimental conditions in body weight (BW), urine specific gravity (USG), and urine color compared to pre‐travel (*p* > 0.05). There was a large effect size for body weight after 4‐h (*d* = 1.66) and a moderate effect size after 2‐h (*d* = 0.81) of air travel.

### 3.4 Perceptual Measures

No significant differences were found in tension, anger, confusion, and depression across conditions (*p* < 0.05).

## 4 Discussion

The present study investigated the impact of short‐haul air travel of different durations on football‐specific performance, sleep patterns, and physiological indices. The principal findings are: (i) football‐specific performance (endurance, sprint, power‐related measures, and technical skills) was impaired following short‐haul air travel, regardless of flight duration; (ii) total sleep time was significantly reduced on pre‐travel nights; (iii) following 4‐h short‐haul air travel, both autonomic and immune function were impaired on the day of flight as well as the day after the travel time.

Consistent with the initial hypothesis, it was shown that the total distance covered during the Hoff test and blood lactate were reduced by approximately 6% (percentage Δ change) and approximately 2 mmol.L^−1^ (absolute Δ change) respectively, in post‐traveling compared to the baseline condition. The current results are opposed to those observed by Fowler et al. who showed unchanged performance in the Yo‐Yo intermittent recovery test after a 5‐h simulated travel in physically active individuals compared to pre‐flight values [7]. It is underlined however, that the participants' traits (physically active individuals vs. highly‐trained football players) and the type of testing were different in the present compared to Fowler's study and this may account for the different results between studies. In addition, there was no lack of sleep in the participants before the flight [7].

Besides the impairment in endurance performance, a significant decay in sprint (i.e., 10 m, RAST) and CMJ was also observed post‐travel. Specifically, both sprinting performance and jumping ability were similarly suppressed post‐travel compared with baseline and control trials, regardless of flight duration. Our findings are in contrast with previous studies, indicating that generic and/or competition performance parameters remain unchanged following short‐haul air travel [5, 7, 20]. For instance, two studies employing either simulated [7] or actual [5] air travel showed unaltered CMJ values after short‐haul flights. The contrasting findings are presumably related to the differences in travel settings as well as to the timing of testing (09:00 PM and 16:00 AM at D + 1) [7, 21].

A significant decline in LSPT was also observed following the simulated air travel indicating that important skills such as passing, dribbling, controlling, and decision‐making are deteriorated post‐travel. Noteworthily, this decrement was more profound following the 4‐h flight. To date, no study has comprehensively examined the influence of air travel in using a sport‐specific technical test such as the LSPT. Our findings may align with the study by Bishop et al., who showed a negative impact of short‐haul traveling (~5‐h) on competition performance [22]. The authors reported no significant change in the point difference between short‐haul and long‐haul flights. They indicated that team performance tends to decline as the distance traveled increases. However, other travel factors, such as sleep disturbances and conditions of somatic idleness, may also negatively impact performance. Equivocal results exist in the literature, with some studies reporting negligible or no effects [5, 20] and others a negative impact on competition performance following short‐haul air travel [22].

The performance deterioration presently observed is likely linked to sleep loss. Indeed, we observed that the total sleep time the night before the flight was shorter by approximately 110 min than D−1, which is due to the early‐morning awakening for the scheduled flight. Similarly, truncated sleep duration (approximately 126 min) the night before the departure was reported for Australian football players, who had to wake up early for a morning flight at a similar departure time to us [1]. However, sleep quality did not change in our study, and sleep quantity was unaffected during the rest days, which is in accordance with the findings of Fowler et al. [1]. In contrast, Richmond et al. reported decreased perceived sleep quality after SHAT [20], although the actigraphy‐based measures, remained unaffected. It is well documented that insufficient sleep can lead athletes to significant physical performance impairments [23, 24]. In particular, Craven et al. highlighted the negative impact of sleep loss on next‐day exercise performance in different physical attributes, including endurance, power, and strength [24]. In addition, several investigations have shown that this influence may be greater when individuals experienced earlier than normal awakening, and when exercise performance was conducted in the PM [24]. These observations suggest that the sleep disruption than the other demands of short‐haul air travel per se may have an adverse impact on football‐specific performance.

Similarly to the reduced aerobic performance recorded in the present participants of the present study, Mougin et al. showed that 3 h of sleep loss the day before action decreased maximal oxygen uptake and increased submaximal heart rate [25]. In our study average heart rate in Hoff's test was similar to control values in both experimental flights, but the total work performed was lower. It appears that sleep disruption [1, 26] can elevate physiological stress, thus resulting in increased sympathetic [27] and cardiovascular activity [26, 27], which can lead to high blood pressure [28]. Assuming that this lack of sleep and the blood pressure elevation persist during exercise, individuals' peak blood pressure will be reached prematurely, and exercise will terminate earlier [29], as it also occurs in our study during the Hoff test. The overactivation of the sympathetic system due to sleep deprivation is further supported [30] by the immunosuppressive response (sIgA drop) found during both flights and 1 day afterwards. Further compounded, there is evidence that a decrease in sIgA might be related to the risk of upper respiratory tract infection [31, 32], an incidence associated with traveling [33]. It is noted that the hallmark of sympathetic activity, namely HRV, has not been greatly investigated, but in the present study was increased only in the 4‐h simulated flight. Likewise with our 2‐h flight, Dranitsin et al. observed no changes in LnRMSSD during the first 3 days following prolonged air travel, with impairments noted only on the fourth and fifth day [34]. Unfortunately, we did not collect data for so long to trace this sympathetic activity oscillation related to traveling. It appears that people suffering from lack of sleep undergo psycho‐physical swings caused by the opposing simultaneous effects of sympathetic hyperactivity (sleep deprivation) and the strong desire to go awake (parasympathetic activation). This may have caused no differences found in psychological state and salivary cortisol across experimental conditions. Similarly, Fowler et al. found no effect on the concentration of salivary cortisol levels following a simulated domestic flight [7]. Furthermore, the present results are consistent with previous studies showing a minor impact of SHAT on muscle soreness, alertness, and stress [5, 6, 7]. It should be noted, however, that the lack of significance between experimental conditions, in cortisol and mood state is in discordance with the literature^37^ and may be, also, due to low instrument sensitivity along with the small number of participants. The response of these variables is known to be associated with significant variability. Our participants, contrary to other studies [5, 6, 7, 20, 22] were a small number of fit soccer players who were used to traveling and lack of sleep.

Sleep deprivation, as occurred in our short‐haul traveling, is associated with altered brain activity in regions related to motor control and alertness^38^ probably affecting tryptophan metabolism, which is involved in sleep regulation and the increase of the perception of central fatigue. In turn, this mechanism would explain the recorded decrease in sprint anaerobic run and countermovement jump. The reduction of anaerobic performance shown in the present study could also be related to an increase in co‐activation of antagonist muscles to running or jumping which is triggered by sleep deprivation [35].

In conclusion, in accordance with our initial assumption, we found that football‐specific performance was decreased after both short‐haul air travels, without being worse, as we initially hypothesized, in the 4‐h flight. Soccer players undergoing short traveling are expected to have diminished aerobic and anaerobic performance, explosiveness of lower extremities, and accuracy of passing and dribbling the ball. Club administrators should not book early‐morning air flights to prevent lack of sleep in their athletes the night before traveling. An increased autonomic stress was observed in soccer players only after the 4‐h simulated flight, whereas immune activity was reduced during the flight days and 24 h after both flights, jeopardizing their health to conduct communicable disease control.

Due to the limited information available, further research is needed to isolate the sleep deprivation factor and investigate the performance decline and the psychophysiological responses after short‐haul flights of varying lengths.

## 5 Limitations, and Perspective

The present study investigated the impact of short‐haul air travel on football players' welfare, utilizing sensitive testing and practical football‐specific measurements within a realistic football scenario. Our findings highlighted physiological alterations and performance deterioration due to travel fatigue. It appears, however, that these limitations are probably due to sleep loss occurring the night before the scheduled flight, rather than air travel logistics per se (e.g., mild hypoxia, cabin discomfort). As the morning flights were organized in the present study, as in most real‐life scenarios, future research should investigate whether different flight times during the day affect sport‐specific performance in real‐life football settings.

## Conflicts of Interest

The authors declare no conflicts of interest.

## Data Availability

The data that support the findings of this study are available from the corresponding author upon reasonable request.
